# Community-based prevalence and associated factors of sarcopenia in the Vietnamese elderly

**DOI:** 10.1038/s41598-023-50979-4

**Published:** 2024-01-02

**Authors:** Lan-Anh Thi Pham, Binh Thanh Nguyen, Dao Tieu Huynh, Binh-Minh Le Thi Nguyen, Phuong-Anh Nhat Tran, Tam Van Vo, Hy-Han Thi Bui, Truc Thanh Thai

**Affiliations:** 1 Department of Nutrition and Food, University of Medicine and Pharmacy at Ho Chi Minh City, 217 Hong Bang Street, Ward 11, District 5, Ho Chi Minh City, Vietnam; 2 Department of Medicine and Pharmacy, Tra Vinh University, 126 Nguyen Thien Thanh Street, Ward 5, Tra Vinh City, Tra Vinh Province Vietnam; 3 Faculty of Public Health, University of Medicine and Pharmacy at Ho Chi Minh City, 217 Hong Bang Street, Ward 11, District 5, Ho Chi Minh City, Vietnam; 4 Laboratory Department, University Medical Center Ho Chi Minh City, 215 Hong Bang Street, Ward 11, District 5, Ho Chi Minh City, Vietnam

**Keywords:** Health care, Medical research, Risk factors

## Abstract

Sarcopenia, a condition characterized by muscle mass decline, is one of the leading health problems in the elderly. This study determined the rate of sarcopenia according to criteria by Asian Working Group for Sarcopenia (AWGS) and related factors in elderly people. A community-based cross-sectional study was conducted in 632 people aged 60 years or over in Ho Chi Minh City. Data were collected through a predefined questionnaire and direct measurement. Sarcopenia was identified based on the Inbody 770 machine and AWGS criteria. The prevalence of sarcopenia was 32.0%. Participants with advanced age, low education, unemployment, low level of family economics and frailty were more likely to have sarcopenia. Among these, frailty had the highest impact on sarcopenia, with significantly higher odds of having sarcopenia found in participants with pre-frailty (OR = 4.80, 95% CI 2.75–8.38, p < 0.001) and frailty (OR = 21.16, 95% CI 8.96–49.97, p < 0.001). In contrast, BMI was inversely associated with sarcopenia. Sarcopenia is prevalent in the Vietnamese elderly. Apart from social demographic characteristics including age, education, employment and family economic status, frailty appeared to be an important risk factor. Early screening, referral, and treatment of sarcopenia among the elderly having a high risk of sarcopenia are recommended.

## Introduction

Sarcopenia is a common health issue among elderly individuals and is characterized by a decrease in muscle mass and a consistent decline in functional capacity^1^. The main causes of sarcopenia are advanced age, low physical activity, multiple concurrent chronic diseases, inadequate energy and/or protein intake, such as malabsorption disorders, gastrointestinal disorders, or medication-induced loss of appetite^2^. The prevalence of muscle loss in elderly individuals is significant among the global population. According to a meta-analysis of 35 studies worldwide, the pooled prevalence of sarcopenia in people over 60 years old is 10%^3^. Based on the estimated prevalence rate, there are currently over 50 million people suffering from sarcopenia, and this condition will affect over 200 million people in the next 40 years^4^. Furthermore, sarcopenia increases the risk of falls, injuries, reduced daily functioning, hospitalization, and mortality^5–7^.

Age-related decline in muscle mass is often diagnosed late because it is considered to be a part of the normal aging process^8^. According to findings of previous studies worldwide, an adult who reaches the age of 50 experiences a gradual decline in muscle mass at an average speed of 1–2% per year, and muscle strength decreases by approximately 1.5% per year^9–11^. In addition to age, malnutrition is closely associated with a decline in muscle mass among older individuals^2^. Malnutrition is one of the main physiological causes of muscle atrophy. The simultaneous presence of these two conditions significantly affects the well-being, mobility, and quality of life of the elderly^12^. On the other hand, other factors considerably contribute to the decline in muscle mass among elderly individuals, such as chronic diseases, body mass index (BMI), lifestyle, and frailty^13,14^. The combination of sarcopenia and its related factors contributes to severe consequences for old adults, including a decline in quality of life, adverse health effects, increased health care costs, and higher rates of hospitalization and mortality^12^.

To date, there is no global consensus on the standardized diagnostic criteria for sarcopenia. Different associations worldwide have varying standards for diagnosing this condition. Among these, the most commonly utilized criteria in both research and clinical practice are the Asian Working Group for Sarcopenia (AWGS)^15^, the European Working Group on Sarcopenia in Older People (EWGSOP)^2^, and the National Institutes of Health (NIH) in the United States^16^. In 2019, the AWGS proposed a diagnostic algorithm based on data from Asian populations, adjusting to anthropometric, cultural, and lifestyle-related differences compared to their Western counterparts. For instance, Asian individuals often have relatively smaller body sizes, higher levels of adiposity, and lead less mechanized but more physically active lifestyles^15^.

Although many studies have reported the prevalence and associated factors of sarcopenia in the elderly globally, there has been limited research conducted on this condition specifically in the Vietnamese population. Findings from previous studies in other countries may not be relevant for Vietnamese population because sarcopenia is relatively population dependent. The anthropometric, cultural, and lifestyle-related differences can lead to different prevalence and manifestation of sarcopenia in specific populations. In Vietnam, the proportion of older adults is increasing, gradually becoming a dominant demographic group^17^. In Ho Chi Minh City (HCMC), one of the largest cities in Vietnam, the proportion of old adults is relatively high, accounting for approximately 7–8% of the total population^18^. The average life expectancy of the residents in this city is also rising, at about 76.6 years according to 2019 statistics^19^. Therefore, it is crucial to assess the health status of the elderly population to implement timely interventions and measures to maintain optimal health. This study determined the prevalence of sarcopenia according to the AWGS criteria among elderly adults and related factors.

## Methods

### Background and study population

A community-based cross-sectional study was conducted from April to July 2022 on the elderly living in District 8, HCMC, Vietnam. In Vietnam, all the elderly are managed by the Elderly Association of each district, where social and medical support is provided when needed. In this study, the participants were selected from the management list of the elderly provided by the Elderly Association of District 8, consisting of individuals aged at least 60 years. We excluded those who had any of the following conditions affecting their participation in this study: communication disability, psychiatric diseases or intellectual disorders, mobility limitation, and severe Parkinson’s disease.

The sample size was calculated by estimating the proportion of patients with sarcopenia. The parameters used in the sample size calculation included a type 1 error probability of 0.05, a marginal error of 0.05, a design effect of 2, and an estimated proportion of sarcopenia in the elderly from a previous study of 0.278^20^. In addition, we anticipated a potential refusal rate of approximately 10%. Therefore, a minimum of 680 elderly individuals was needed for this study.

### Methods

A multi-stage sampling approach was used to recruit the elderly. In District 8, there are 16 administrative wards. We randomly selected 5 wards. From the sampling frame obtained from the Elderly Association of District 8, individuals were randomly chosen. Subsequently, a list of eligible individuals was compiled. Invitation letters and consent forms were then sent to invite them to participate in the study. During the study, each participant was directly interviewed using a questionnaire. Body measurements were also taken at the designated research site in each ward.

### Measurement

The interview questionnaire included information on personal and social characteristics, nutritional status, comorbidities, fatigue, physical activity intensity, and frailty. Personal and social characteristics included age group (60–69; 70–79; and ≥ 80), gender (male/female), ethnicity (Kinh or Others), education level (< high school and ≥ high school), employment (yes/no), co-habitation (with relatives and alone), low family economics (yes/no, defined as a monthly income of less than 1.5 million VND per individual in rural areas and less than 2.0 million VND per individual in urban areas), smoking (yes/no)and drinking alcohol (yes/no). We also recorded information about hospitalization and fall in the past 12 months (yes/no), polypharmacy (yes/no, defined as the use of more than 5 types of medications), and multiple comorbidities (yes/no, assessed using the Charlson comorbidity index^20^, which assigns scores to various medical conditions and a total score of ≥ 2 indicates the presence of multiple comorbidities). Body Mass Index (BMI) was calculated based on weight and height and was subsequently categorized into three groups, including underweight (< 18.5), normal (18.5–< 23.0) and overweight/obese (≥ 23) for those aged < 65 years^21^ and underweight (< 23), normal (23–< 30) and overweight/obese (≥ 30) for those aged ≥ 65 years^22^.

Frailty was evaluated based on Cardiovascular Health Study (CHS) index via presenting of criteria such as unintentional weight loss (of ≥ 5% of body weight in prior year), self-reported exhaustion, weakness (determined by utilizing a Camry EH101 device), slow walking speed, and low physical activity. Frailty was defined as having three or more of the mentioned criteria, while having one or two criteria was considered pre-frail. If none of the criteria was present, the individual was classified as non-frail^23^. Malnutrition was evaluated by using the Mini Nutritional Assessment (MNA) scale and was categorized into three groups, including well-nourished (≥ 24 points), at risk of malnutrition (from 17 to 23.5 points), and malnourished (< 17 points)^24^. The level of sarcopenia was assessed based on the Asian Working Group for Sarcopenia (AWGS) criteria established in 2019^15^. Muscle mass (kg) was evaluated using Inbody 770, a bioelectrical impedance analysis (BIA) device. Muscle strength was assessed using a Camry EH101 device.

### Statistics

The study data were entered and stored using Epi-data 3.1 software and analyzed using Stata 14.0. Descriptive statistics, including frequencies and percentages, were used for categorical variables. For quantitative variables, if the data had a normal distribution, mean and standard deviation were used. If the distribution was skewed, the data were described using median and interquartiles. Chi-square tests and logistic regression were employed to determine factors associated with sarcopenia. Statistically significant variables from simple logistic regression were used in multiple logistic regression. Non-significant variables from the initial multiple logistic regression were excluded and the final model was fitted using the other variables. The likelihood ratio test was used to compare the initial model and the final model. For the final model, goodness of fit was evaluated using the Pearson Chi-Squared test and the Hosmer–Lemeshow Chi-Squared test. Collinearity was assessed using variance inflation factor (VIF) and tolerance statistics. A significance level of p < 0.05 was used (Supplementary Information).

### Ethics

All study procedures were performed in accordance with the relevant guidelines and were approved by the Ethics Committee of the University of Medicine and Pharmacy at HCMC (No. 11/DHYD-HDDD dated January 10, 2022). Informed consent was obtained from all participants.

## Results

During the study period, among 680 elderly individuals approached, 632 agreed to participate and were included in the data analysis. Among these, most were female (68.4%), aged 60–69 years (62.3%), Kinh ethnic (87.5%) and had an education level of below high school (66.5%). Most of the elderly in the study were unemployed (75.8%), living with relatives (94.8%). Most participants reported no smoking (88.1%) or drinking alcohol (94.9%). A total of 202 elderly (32.0%) were identified to have sarcopenia. A significantly higher prevalence of sarcopenia was found among elderly with advanced age, low education level, unemployment, and low-income families. There was no significant association between sarcopenia and gender, ethnicity, co-habitation, smoking and drinking alcohol (Table 1).

**Table 1:** Socio-demographic characteristics and their association with sarcopenia.

	Total *N* = *632* *n (%)*	Sarcopenia		
		Yes *(n* = *202; 32.0%)* *n (%)*	No *(n* = *430; 68.0%)* *n (%)*	p
Age group (year)				
60–69	394 (62.3)	90 (44.6)	304 (70.7)	< 0.001
70–79	192 (30.4)	80 (39.6)	112 (26.0)	
≥ 80	46 (7.3)	32 (15.8)	14 (3.3)	
Gender				
Male	200 (31.6)	69 (34.2)	131 (30.5)	0.352
Female	432 (68.4)	133 (65.8)	299 (69.5)	
Ethnicity				
Kinh	553 (87.5)	175 (86.6)	378 (87.9)	0.652
Others	79 (12.5)	27 (13.4)	52 (12.1)	
Education level				
< High school	420 (66.5)	155 (76.7)	265 (61.6)	< 0.001
≥ High school	212 (33.5)	47 (23.3)	165 (38.4)	
Employment				
Yes	153 (24.2)	29 (14.4)	124 (28.8)	< 0.001
No	479 (75.8)	173 (85.6)	306 (71.2)	
Co-habitation				
With relative	599 (94.8)	187 (92.6)	412 (95.8)	0.088
Alone	33 (5.2)	15 (7.4)	18 (4.2)	
Low family economics				
Yes	84 (13.3)	43 (21.3)	41 (9.5)	< 0.001
No	548 (86.7)	159 (78.7)	389 (90.5)	
Smoking				
Yes	75 (11.9)	31 (15.3)	44 (10.2)	0.064
No	557 (88.1)	171 (84.7)	386 (89.8)	
Drinking alcohol				
Yes	32 (5.1)	9 (4.5)	23 (5.3)	0.633
No	600 (94.9)	193 (95.5)	407 (94.7)	

Table 2 presents health-related factors of the elderly participated in this study. About 11.9% of participants reported having been hospitalized in the past 12 months and 9.3% had a history of fall in the past 12 months. Most of participants were classified as pre-frailty (58.2%), did not use polypharmacy (92.1%), did not have multiple comorbidities (79.3%) and had a BMI classification as normal (45.4%). Based on the MNA, the prevalence of at risk of malnutrition and malnourished was 29.1% and 4.0%, respectively. Among these factors, a history of hospitalization, a history of falls, frailty, malnutrition, and BMI were significantly associated with sarcopenia (Table 2).

**Table 2:** Other characteristics and their associations with sarcopenia (N = 632).

	Total *N* = *632* *n (%)*	Sarcopenia		
		Yes *(n* = *173; 27.4%)* *n (%)*	No *(n* = *459; 72.6%)* *n (%)*	p
Hospitalization in the past 12 months				
No	557 (88.1)	167 (82.7)	390 (90.7)	0.004
Yes	75 (11.9)	35 (17.3)	40 (9.3)	
Fall in the past 12 months				
No	573 (90.7)	174 (86.1)	399 (92.8)	0.007
Yes	59 (9.3)	28 (13.9)	31 (7.2)	
Frailty				
No frailty	201 (31.8)	19 (9.4)	182 (42.3)	< 0.001
Pre-frailty	368 (58.2)	134 (66.3)	234 (54.4)	
Frailty	63 (10.0)	49 (24.3)	14 (3.3)	
Polypharmacy				
No	582 (92.1)	186 (92.1)	396 (92.1)	0.995
Yes	50 (7.9)	16 (7.9)	34 (7.9)	
Multiple comorbidities				
No	501 (79.3)	171 (84.7)	330 (76.7)	0.022
Yes	131 (20.7)	31 (15.3)	100 (23.3)	
Malnutrition				
Normal nutritional status	423 (66.9)	100 (49.5)	323 (75.1)	< 0.001
At risk of malnutrition	184 (29.1)	83 (41.1)	101 (23.5)	
Malnutrition	25 (4.0)	19 (9.4)	6 (1.4)	
Body Mass Index (kg/m^2^)				
Normal	287 (45.4)	73 (36.1)	214 (49.8)	< 0.001
Underweight	207 (32.8)	119 (58.9)	88 (20.5)	
Overweight/obese	138 (21.8)	10 (5.0)	128 (29.8)	

Factors significantly associated with sarcopenia in Table 1 and 2 were further analyzed using univariable and multiple logistic regression in Table 3. In this study, the final model fitted the data well with no significant collinearity detected. Participants with advanced age, low education, unemployment, low level of family economics and frailty were more likely to have sarcopenia. Among these, frailty had the highest impact on sarcopenia, where significantly higher odds of having sarcopenia found in participants with pre-frailty (OR = 4.80, 95% CI 2.75–8.38, p < 0.001) and frailty (OR = 21.16, 95% CI 8.96–49.97, p < 0.001). Moreover, BMI was inversely associated with sarcopenia.

**Table 3:** Multivariable regression analysis between factors associated with sarcopenia (N = 632).

	Simple logistic regression		Multiple logistic regression	
	OR (95% CI)	p	OR (95% CI)	p
Age group				
60–69	1		1	
70–79	2.41 (1.66–3.50)	< 0.001	1.40 (0.90–2.17)	0.138
≥ 80	7.72 (3.95–15.10)	< 0.001	2.82 (1.26–6.32)	0.012
Education level				
< High school	2.05 (1.40–3.00)	< 0.001	1.72 (1.08–2.73)	0.022
≥ High school	1		1	
Employment				
Yes	1		1	
No	2.42 (1.55–3.77)	< 0.001	1.85 (1.10–3.12)	0.021
Low family economics				
No	1		1	
Yes	2.57 (1.61–4.09)	< 0.001	1.85 (1.03–3.29)	0.038
Hospitalization in the past 12 months				
No	1			
Yes	2.04 (1.25–3.33)	0.004		
Falls in the past 12 months				
No	1			
Yes	2.07 (1.21–3.56)	0.008		
Frailty				
No frailty	1		1	
Pre-frailty	5.49 (3.27–9.21)	< 0.001	4.80 (2.75–8.38)	< 0.001
Frailty	33.53 (15.69–71.62)	< 0.001	21.16 (8.96–49.97)	< 0.001
Malnutrition				
Normal nutritional status	1			
At risk of malnutrition	2.65 (1.84–3.83)	< 0.001		
Malnutrition	10.23 (3.98–26.31)	< 0.001		
Body Mass Index (kg/m^2^)				
Normal	1		1	
Underweight	3.96 (2.70–5.81)	< 0.001	3.32 (2.15–5.11)	< 0.001
Overweight/obese	0.23 (0.11–0.46)	< 0.001	0.25 (0.11–0.53)	< 0.001

Goodness of fit assessment for the final model: Pearson Chi-Squared p-value = 0.625; Hosmer–Lemeshow Chi-Squared p-value = 0.907. Collinearity diagnostics for the final model: VIF = 1.03–1.11, Tolerance = 0.90–0.97.

## Discussion

This study was among the first community-based studies to investigate sarcopenia among the elderly in Vietnam. Based on the AWGS, we found that the prevalence of sarcopenia was high, with more than one-fourth of the elderly classified as having this condition. Independent factors associated with sarcopenia included advanced age, low education, unemployment, low-income family, and frailty.

Sarcopenia is a health condition relating to the decline of muscle mass in combination with the low skeletal muscle strength and the presence of low physical activity^15^. Since these characteristics are age related, sarcopenia is common and one of the major health problems in the older population^2,3^. The high prevalence of sarcopenia observed in our study is consistent with the result of a study conducted on a similar population in Korea^20^, but is higher than that of studies performed in China and lower than that of several studies conducted in the United States^13,25,26^. The variation in the prevalence of sarcopenia, as reported in our study and others, is due to various factors. First, differences in lifestyles and cultures among study populations can result in different prevalences of sarcopenia. Second, the inclusion of certain characteristics of study participants, such as age groups, with or without any specific comorbidity may also affect the estimate of prevalence of sarcopenia. Third, the use of diagnostic standards and measuring devices such as BIA or DXA also leads to differences in the prevalence of sarcopenia^13,25,26^. Although the results in different study populations may not be absolutely comparable as explained above, the high prevalence of sarcopenia consistently reported in our study and others indicates that sarcopenia is an important health issue among the elderly and warrants further investigation and intervention.

In resource-limited settings, the high prevalence of sarcopenia may lead to another burden for both individuals and health care system. Therefore, targeting those who are more vulnerable through their higher risk of having sarcopenia may be more beneficial. In our study, the associated factors of sarcopenia including advanced age, low social economic status (i.e., low education, unemployment, low family economic status) and health status (i.e., frailty, overweight) were found to be important. Our findings are supported by data from previous studies in various populations such as those in Thailand (2020) and China (2016)^14,27^. However, in our study, the role of frailty in sarcopenia was more pronounced than that reported in Thailand^14^. Among a total of 330 outpatients aged over 60 years, the study in Thailand reported 34% pre-frailty and no frailty while such figures in community-based sample in our study were 58.2% and 10.0%. Importantly, in our study those with pre-frailty and frailty were much more likely to have sarcopenia compared to that reported in the study in Thailand. Moreover, an inverse association between BMI and sarcopenia was observed in previous studies^13^.

Some implications can be learned from our study. First, a high prevalence of sarcopenia in a large community-based sample means that sarcopenia is under-diagnosis and under-treated in the elderly in Vietnam. Based on the consistently high prevalence reported in the literature review, sarcopenia may also be a global health problem in this vulnerable population. Early and routine screening and referral, followed by optimal treatment can be beneficial for the elderly and limit the burden of sarcopenia on the health care system. Second, because the associated factors of sarcopenia may be context dependent, those reported in previous studies such as alcohol use^13^ may not be relevant in Vietnam. To optimize resources in healthcare, the associated factors in our study can be used to target those who had higher risk and thus more vulnerable to sarcopenia. More studies are also recommended to identify risk profiles for the elderly in different settings. Third, our data collection was conducted through the Elderly Association at each ward within the district. In Vietnam, the role of this association is to organize social activities for the elderly and to provide a wide range support including practical support, financial support, and medical support. Community intervention such as health education and promotion to improve the awareness of sarcopenia integrated and led by this organization may be beneficial and acceptable.

Our study has some strengths including the relatively large sample size of the elderly in the community, the use of standardized equipment and criteria to assess variables and outcomes. However, there are still some limitations in our study. First, this was a cross-sectional study and thus the underlying causes and causal relationships between factors reported with sarcopenia require further investigation. Second, despite the large sample size, our study participants were from one district within a big city which may affect the generalizability of the study findings. Third, sarcopenia can be affected by various factors, many of which were not included in our study such as physical activity, nutrition intake and the duration of living with comorbidity. Further studies including these factors and others can clarify the manifestation of sarcopenia in the elderly.

## Conclusion

Sarcopenia is prevalent in the Vietnamese elderly. Apart from social demographic characteristics such as age, education, employment and family economic status, frailty appeared to be an important risk factor. Early screening, referral, and treatment of sarcopenia among the elderly who have a high risk of sarcopenia are recommended.

### Supplementary Information

Supplementary Information.

## Data Availability

Collected and analyzed data during the study are available from the corresponding author upon reasonable request.
